# Bridging Data Gaps: Predicting Sub-national Maternal Mortality Rates in Kenya Using Machine Learning Models

**DOI:** 10.7759/cureus.72476

**Published:** 2024-10-27

**Authors:** Hellen Muringi Mwaura, Timothy Kelvin Kamanu, Benard W. Kulohoma

**Affiliations:** 1 Department of Biochemistry, University of Nairobi, Nairobi, KEN; 2 Department of Mathematics, University of Nairobi, Nairobi, KEN; 3 Centre for Biotechnology and Bioinformatics, University of Nairobi, Nairobi, KEN; 4 Infectious Disease, International AIDS Vaccine Initiative (IAVI), Nairobi, KEN

**Keywords:** demographic and health survey, indicators of maternal mortality, kenya, machine learning models, maternal mortality, maternal mortality ratio, sub-national

## Abstract

Introduction

Maternal mortality remains a critical global health issue, with ongoing efforts to reduce its incidence as part of international health priorities. Kenya, a sub-Saharan country that has a disproportionate number of maternal mortality is likely to miss this target unless evidence-based interventions are deployed. The paucity of reliable maternal health data calls for the development of alternative predictive models to complement the impaired civil registration system and the aperiodic national surveys.

Methods

We utilized DHS surveys from several Sub-Saharan African countries to estimate parameters for predicting Kenya’s maternal mortality rate (MMR) in the absence of recent Kenya Demographic and Health Survey (KDHS) data. We developed a multiple linear regression model using supervised machine learning using the R-programming suite. Our model leverages machine learning techniques to analyze regional trends and predict sub-national MMR variations. We then applied the model to predict MMR for Kenyan counties using the data for the KDHS 2022 survey.

Results

Using Pearson’s correlation, we observed a significant positive correlation between MMR and total fertility (r = 0.32, p = 0.025) and a significant negative correlation between MMR and maternal age at first birth (r = -0.40, p = 0.005). Additionally, a significant correlation was observed with the cumulative percentage of mothers attending post-natal clinics, the prevalence of thinness (r = 0.77, p < 0.001), HIV infection in women (r = 0.20, p = 0.164), and physical violence during pregnancy. The model estimate of national MMR in 2022 was 367 deaths per 100,000 live births, ranging from 49 deaths per 100,000 live births in Kisii County to 1794 deaths per 100,000 live births in Turkana County.

Conclusion

Although MMR in Kenya displayed a general downward trend, our model’s estimates for DHS 2022 indicate an increase compared to the 2019 National Census and Housing Survey estimate of 355 deaths per 100,000 live births. This rise may be attributed to COVID-19-related maternal deaths during the same period. The integration of predictive models to inform interventions and resource allocation could play a crucial role in enhancing maternal healthcare outcomes in Kenya.

## Introduction

Maternal mortality refers to the deaths of mothers as a result of complications during pregnancy, childbirth, and the initial two months postpartum exacerbated by complications related to pregnancy or the subsequent management procedures; excluding accidental or incidental factors [1-3]. It is a global health concern that is estimated to occur every two minutes and is therefore targeted in the global Sustainable Development Goals (SDG 3.1) [1,4].

The number of global maternal deaths in 2020 was 287,000, which depicted a sub-optimal rate of reduction from the 309,000 maternal deaths in 2016 when the SDG was implemented [1]. The global maternal mortality ratio (MMR) in 2020 was estimated at 223 deaths per 100,000 live births; a decline from 227 in 2015 [2]. However, this figure remains higher than the worldwide target of 140 deaths per 100,000 live births set for the year 2030 [1]. Maternal health disparities are evident across global regions, with developing nations accounting for 90% of these fatalities. Countries in Sub-Saharan Africa contributed up to 80% of all maternal deaths in the year 2020 [1,3].

The MMR in Kenya has slowly declined from 497 deaths per 100,000 live births in 2009 to 355 deaths per 100,000 live births in 2019. Modeled estimates suggest a further decline to 158.8 deaths per 100,000 live births in 2021 [1]. This reduction remains above the SDG 3.1 target; and Kenya’s national MMR target of less than 70 deaths per 100,000 live births [5]. Concerted efforts at the sub-national level are required to address disparities in maternal health systems within the country. Efforts such as scaling up community-based interventions, improving access to skilled birth attendants, and strengthening referral systems are ongoing to reduce maternal mortality. However, challenges such as poor access to healthcare services in remote areas, inadequate health infrastructure, and a shortage of skilled healthcare workers, continue to hinder progress [6-9]. There is a notable paucity of data on the surveillance and monitoring of maternal mortality due to impaired civil registration systems that have led to exclusive reliance on periodic national surveys. These include the quinquennial demographic health survey (DHS) and the decennial national census, which are also vulnerable to external disruptions as exemplified by the significant delay in releasing the recent 2022 DHS survey due to the unforeseen impacts of the COVID-19 pandemic.

Kenya Demographic and Health Survey (KDHS) surveys do not include subnational MMR due to the underreporting and the infrequent nature of data collation [3]. Subnational MMR estimates are estimated from national census surveys that occur once a decade [7,10]. Predictive tools capable of estimating maternal mortality ratios are required to address this information gap and guide the development and implementation of health policies toward achieving the SDG 3.1 target [3,4]. Predictive models are becoming more commonplace for addressing these gaps [9]. These models are able to infer risk factors well in advance.

We developed a model to predict MMRs in Kenyan counties by leveraging supervised machine learning to overcome limitations caused by a lack of data. The model bridges data gaps created by the infrequent census and DHS surveys. Our findings will highlight targeted interventions aimed at MMR that will enable appropriate resource allocation in Kenyan counties and subsequently accelerate the attainment of SGD 3.1. The model addresses the heterogeneity in maternal health care in Kenya that is not addressed using evidence-based interventions. This study aimed to develop and evaluate a predictive model for maternal mortality rates (MMR) using various socio-demographic and health-related variables.

## Materials and methods

Study design

We employed a retrospective observational study design and utilized a multiple linear regression model to predict maternal mortality ratios (MMR) using a comprehensive dataset derived from Demographic and Health Surveys (DHS). The dataset included over 20 relevant socio-demographic and health-related variables. The use of DHS data was pivotal, as it follows standardized protocols for data collection, anthropometric measurements, and maternal health indicators across multiple countries. This approach not only enabled us to effectively manage the large-scale dataset but also ensured the model's robustness and accuracy in predicting maternal mortality outcomes across diverse settings.

These datasets were from lower-middle-income African countries as guided by the 2022 World Bank classification. These countries have similar profiles in their common cultural practices, endemic tropical infectious diseases, healthcare environments, and socio-economic profiles, and therefore provided a wider dataset for informing the development of a robust predictive model for Kenya by improving the generalizability of the model and therefore provided a wider, harmonized, and controlled dataset, ensuring validity and reliability, to inform the development of a robust predictive model for Kenya and improve the generalizability of the results.

A total of 41 national datasets were included in this study, and include Angola, Benin, Burkina Faso, Burundi, Cameroon, Cape Verde, Chad, Comoros, Congo, the Democratic Republic of the Congo (DRC), Côte d'Ivoire, Equatorial Guinea, Eritrea, Eswatini, Ethiopia, Gambia, Ghana, Guinea, Kenya, Lesotho, Liberia, Madagascar, Malawi, Mali, Mauritania, Mozambique, Niger, Nigeria, Rwanda, São Tomé and Príncipe, Senegal, Sierra Leone, Tanzania, Togo, Uganda, Zambia, and Zimbabwe.

Selection of the surveys

We considered surveys that were conducted after the establishment of the Millennium Development Goals (MDGs) and before the COVID-19 pandemic. Surveys conducted prior to the year 2000 were excluded because of a lot of missing data. Language was not considered as a selection criterion, and reports in multiple languages (French, English, Portuguese, and Spanish) were considered. A total of 102 surveys from 41 African countries were used to develop our model, and this ranged from 1 to 5 surveys per country, with an average number of 3 surveys per country.

Selection of indicators

Twenty indicators of maternal mortality were selected as model variables. These predictive variables take into account that there is an increased liability of maternal death with an increased likelihood of pregnancy, and this is heightened by complications during pregnancy. These variables were categorized as direct indicators, indirect indicators, cultural practices, and infectious diseases. 

Data extraction and management

Data on country names, MMR, and the 20 variables were retrieved from all 102 DHS reports. Missing data was observed in some survey reports, with at least one missing variable in up to 78% of the entire dataset (Table 1). Missing data was imputed in two steps: first, we imputed each variable using national average values, and then we imputed the remaining missing values using an automated linear regression approach. This was done using the “mice’ package in the R-programming suite [11].

**Table 1:** Level of missingness in data variables

Percentage of missingness	Number of variables
0% - 0.9%	5
1% - 10%	9
11% - 20%	2
21% - 30%	1
31% - 41%	1
40% - 50%	1
Total	20

Data cleaning and data pre-processing

Data was encoded on the country name and year column. A threshold of 15% was used for cesarean sections as guided by the WHO guidelines, which suggests that rates between 10-15% are sufficient to address most medical indications for C-sections; Beyond which higher rates may indicate that the procedures are performed for non-medical reasons, such as patient preference or institutional practices, rather than as a response to obstetric emergencies.

A collinearity diagnostic test was performed on the variables to identify and subsequently filter out highly correlated confounding variables to mitigate multicollinearity. The Shapiro-Wilk normality test and Q-Q plot were used to confirm the variables had a normal data distribution. Outliers were identified and subsequently filtered out using a threshold of 3 standard deviations from the mean.

 Model development and fitness test

The model was developed using supervised machine learning using the R-programming suite [11]. The dataset was divided into training and test sets with a split ratio of 0.7. The model was developed using multiple linear regression (lm 4 package)11. The test set was used to validate the model. R-square (R²) and mean squared error (MSE)values were used to estimate the accuracy and magnitude of the model's predictive capability. The model achieved an R² value of 75.5%, indicating that it explained 75.5% of the variance in MMR, and an MSE of 30 deaths per 100,000 live births.

Application of the model to describe sub-national MMR in Kenya

The 2022 DHS survey did not include estimates for national and sub-national MMR; To address this gap, we employed the model to project MMR values using the DHS 2022 dataset and compared these predictions with estimates from the Kenyan 2019 census to provide insights into the current MMR landscape in Kenya.

The model was used to predict MMR for the KDHS 2022 data and the confidence intervals were determined in order to compare the observed values with the predicted values. The predicted MMR values were then compared with the MMR values from the Kenyan 2019 Census data and used to describe the subnational situation in Kenya.

Validity of the model

While dividing data into training set and test set, different split ratios were tested and an optimum was identified at 0.7. We tried different models, such as the generalized estimating equation (GEE) model to the same data, however, they had suboptimal performance compared to the multiple linear regression model, which yielded more robust results in predicting MMR.

The model was trained by five-fold cross-validation and by dividing the data according to sub-regions in Africa. The East African datasets were applied for Kenya and provided the most robust prediction outcomes.

## Results

Descriptive analysis of variables

Descriptive analysis was done on the variables by determining the mean and the standard deviations (Table 2). The MMR ranged from 40 to 1480 deaths per 100,000 live births. The total fertility was expressed as the number of children per woman and ranged from 2.5 to 7.6 with a mean of 5.1 children per woman. The birth intervals refer to the mean number of months since the preceding pregnancy; they are expressed in months and range from 29.2 to 47.1 with a mean of 35.2 months. The median age at first birth was 19.3 years, and the median age at first marriage was 18.9 years, with respective ranges of 16.2 to 23.9 years and 15.5 to 23.7 years.

**Table 2:** Descriptive statistics of the variables

Variable	Mean	Range	Std. Deviation	Variance
Total fertility	5.1	2.5 - 7.6	1	1
Birth intervals (months)	35.3	29.2 - 47.1	3.8	14.2
Median age at first birth (years)	19.3	16.2 - 23.9	1.8	3.2
Median age at first marriage (years)	18.9	15.5 - 23.7	1.6	2.5
Teenage mothers	22.7	4.1 - 43.4	9.7	93.5
Modern contraceptives (married women)	24.2	4.4 - 65.8	17.5	306.7
Modern contraceptives (all women)	20.7	1.5 - 61.5	13.6	184.4
ANC (4+ times)	54.4	10.4 - 99.0	20.6	426
Health facility delivery	56.7	5.3 - 93.1	21	442.3
Distance to health facility	37.9	0.6 - 71.4	13.7	192
Skilled birth attendants	59.7	5.7 - 94.2	19.7	394.5
Percentage of cesarean-section births	6.07	0.4 - 95.8	10.9	118.4
PNC in 48 hours	38.7	1.0 - 87.9	23.3	543.6
Shortness (height <145 cm	2.1	0.2 - 13.1	1.8	3.4
Moderately and severely thin (BMI <17)	4.1	0.0 - 30.1	4	15.9
Overweight and obese (BMI >25)	25.5	4.4 - 99.6	17	288.6
HIV-positive women	9	0.2 - 39.0	9.2	84.3
Violence during pregnancy	7.39	0.2 - 34.0	4.9	24.25
Women's autonomy toward their own health	52.4	12.9 - 96.1	22.4	503.1
Maternal mortality ratio	575.3	40.3 - 1480.7	261.4	68315.7

The antenatal clinic attendance refers to the number of women who attended at least four clinic visits during the previous pregnancy and had a range of 10.4-99% with a mean of 54.4%. Postnatal care clinic attendance refers to the percentage of mothers who received a postnatal check-up from a trained health practitioner within 48 hours after delivery and ranged from 1-87.9%, with a mean of 38.7%. In this study, shortness refers to the percentage of mothers whose height was ≤145cm, with a range of 0.2-13.1% and a mean of 2.1%. Thin mothers (BMI ≤17) ranged from 0-30.1% of the dataset, with a mean of 4.1%, while overweight and obese mothers (BMI >25) ranged from 4.4-99.6%, with a mean of 25.5. Women’s autonomy toward their own health was expressed as the percentage of women who were involved either independently or jointly in making decisions concerning their own health, with a mean of 52.4%, ranging from 12.9-96.1%.

Model description

After model development, we observed that total fertility, birth intervals, age at first birth, prevalence of postnatal care, thinness, prevalence of HIV-infected women, and violence during pregnancy were significant predictors of MMR with p-values less than 0.05 at a 95% confidence interval (Table 3).

**Table 3:** Multiple linear regression model results: coefficients, standard errors, and p-values at 95% confidence intervals p-value <0.05 is significant, and marked with an asterisk (*)

Indicator	Coefficients	Standard-errors	P-values
(Intercept)	-2622.63	459.23	0.00*
Total fertility	112.76	27.23	0.00*
Birth intervals (months)	36.38	6.42	0.00*
Age at first birth	27.13	8.62	0.00*
Post-natal clinic attendance	-5.46	0.99	0.00*
Thinness	33.68	7.40	0.00*
HIV-infected women	15.01	3.78	0.00*
Violence in pregnancy	-6.85	2.38	0.00*
Age at first marriage	43.75	18.96	0.02*
Distance to a health facility	3.05	1.49	0.04*
Cesarean section	-19.11	10.57	0.07
Skilled birth attendants	-2.48	1.39	0.08
Prevalence of FGM	-0.80	0.53	0.14
Health facility delivery	2.05	1.44	0.15
Modern contraception	-1.86	1.72	0.28
Overweight and obese	3.38	3.34	0.31
Shortness	-11.73	14.60	0.42
Teenage mothers	-1.68	2.48	0.50
Women's autonomy toward their own health	-0.35	0.94	0.71
ANC clinic attendance	0.19	1.11	0.86
Contraception in marriage	0.30	1.94	0.88

The model: MMR = 112.7567 (TF) + 36.37513 (BI) + 27.12801 (AB) - 5.45836 (PNC) - 33.68118 (Thin) + 15.00984 (HIV) + -6.84887 (Violence) - 2622.63.

Where: MMR = Maternal mortality ratio, TF = Total fertility, BI = Birth intervals, AB = Age at first birth, PNC - Prevalence of postnatal care, Thin = Prevalence of thinness (<145 cm), HIV - Prevalence of HIV in women, Violence - Prevalence of violence during pregnancy

**Figure 1:**
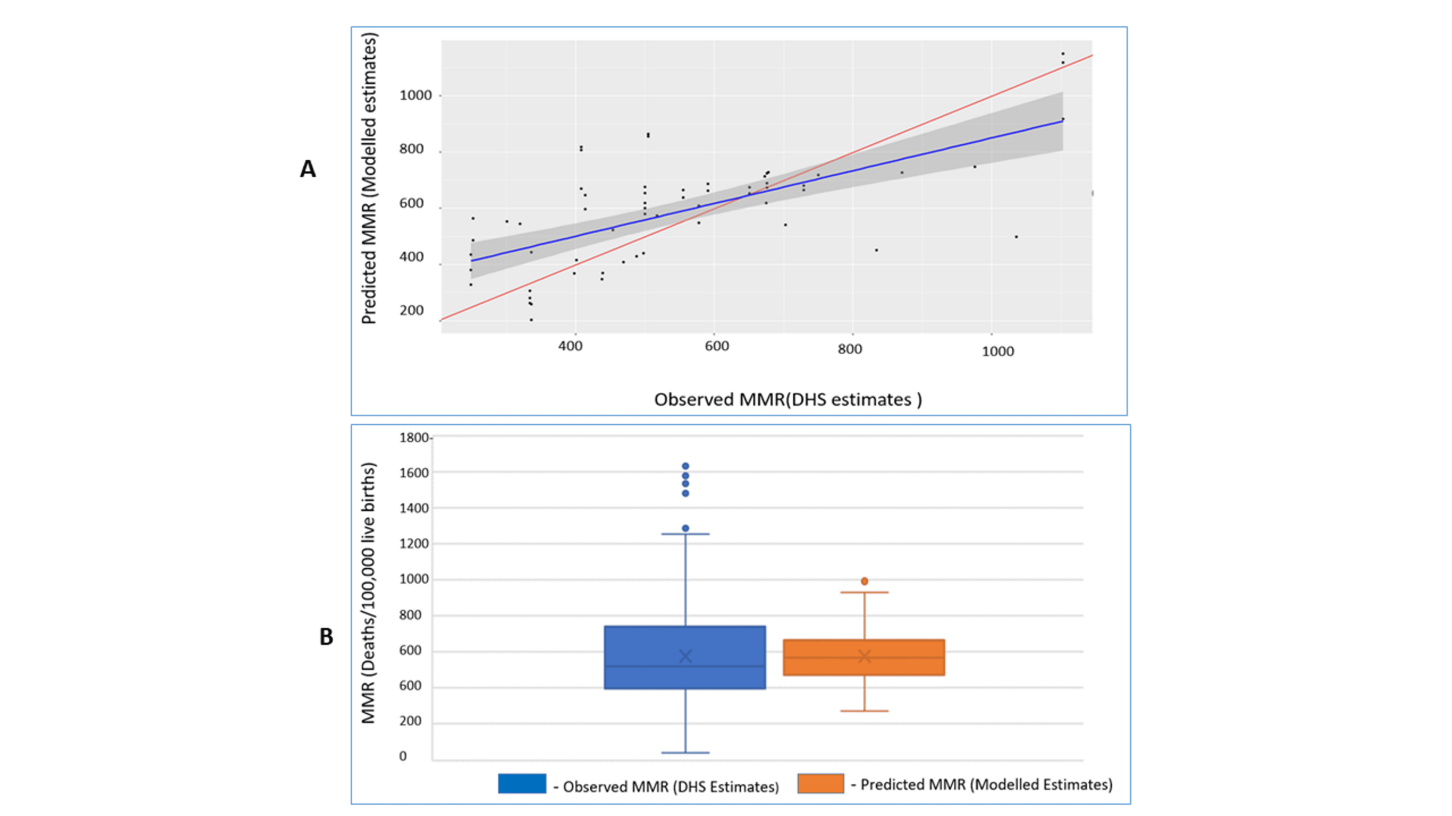
Comparing the DHS estimates of MMR with the model predictions (A) Comparing the model-predicted MMR estimates and the DHS-observed estimates; (B) Comparing the quality of the model estimates with those of the DHS estimates. MMR: maternal mortality rate; DHS: Demographic and Health Survey

We compared observed (DHS estimated) versus predicted MMR values (Figure 1A). The model-predicted values showed a reduced number of outliers compared to DHS estimates, with a narrower interquartile range and a mean closely aligned with the median. The model produced robust predictions with an R-squared value of 0.75 at 95% CI (Figure 1B).

Model estimation of MMR in Kenya using DHS 2022 data

**Figure 2:**
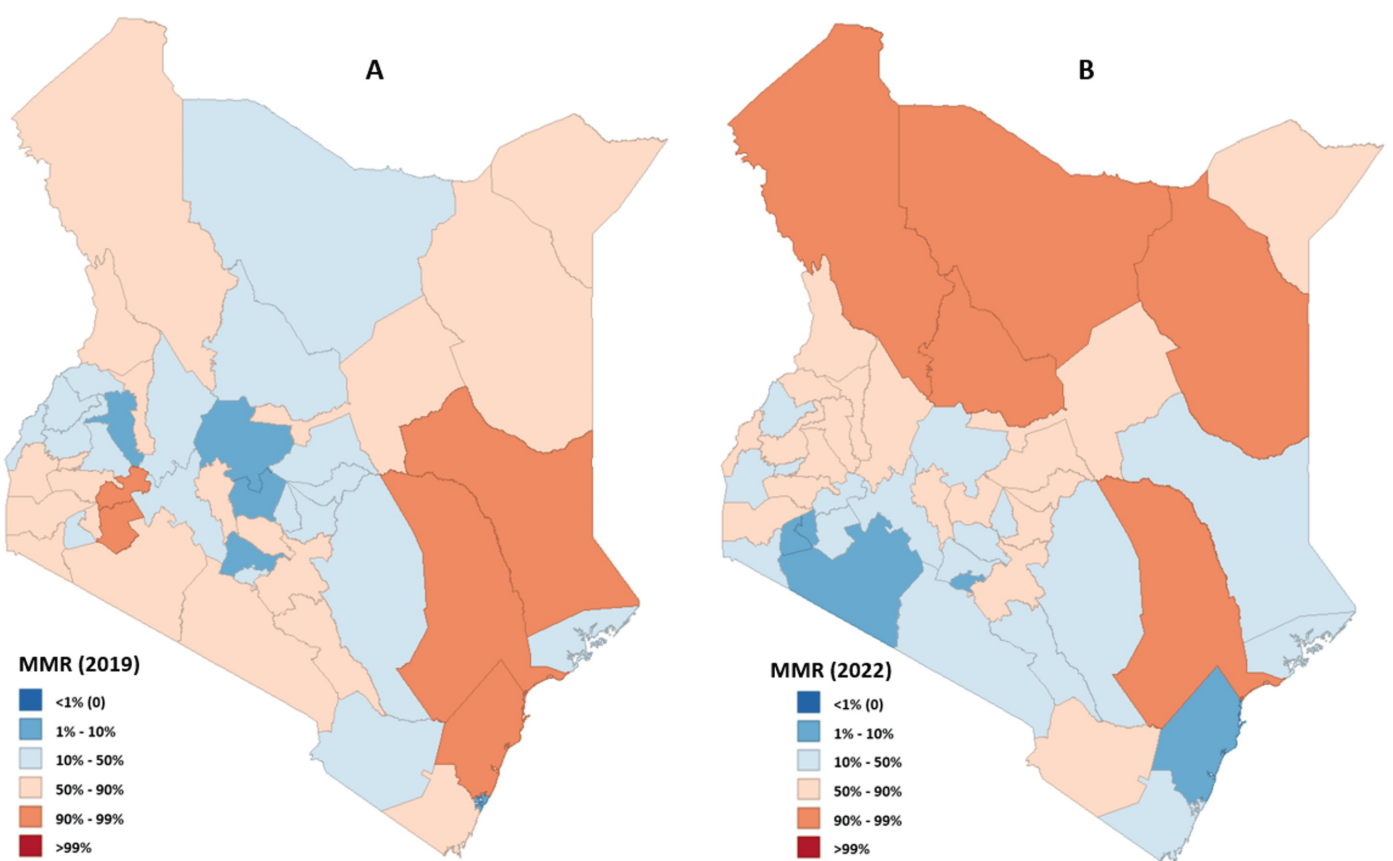
MMR in Kenya by county Source - Image created by the author (Hellen) using GeoDa application version 1.18. (A) Distribution of maternal mortality in Kenya in 2019; (B) Distribution of maternal mortality ratios in Kenyan counties in 2022 MMR: maternal mortality rate

The 2022 Kenya DHS survey did not have national and subnational maternal mortality estimates. We used the developed model to address this information gap. We then compared our findings with those from the 2019 census (355 deaths per 100,000 live births) to establish changes in MMR between the two time intervals. Our model prediction for the 2022 MMR was 367 deaths per 100,000 live births. MMR estimates from the preceding DHS survey in 2014 were 362 deaths per 100,000 live births. Indicating an increase in national MMR values in 2022. We also used the model to predict sub-national level MMR values (Figure 2).

## Discussion

In recent times, health research has witnessed a surge in the adoption of machine learning techniques coupled with artificial intelligence to make inferences about complex health challenges to prepare appropriate interventions [11-13]. These advanced methodologies have also received considerable attention in the development of predictive models that assess the likelihood of complications arising during pregnancy or childbirth [14-16]. These approaches are important for forecasting risk factors associated with maternal morbidity and mortality, enabling strategic interventions to safeguard the well-being of expectant women. There is a gap in crucial information required to guide policy formulation and resource allocation at the national and sub-national levels in Kenya. This underscores the need for exploiting machine learning approaches to address challenges posed by high maternal mortality.

The model implemented in this study takes into account that increased liability of maternal death is associated with the prevalence of infectious diseases, social demographics, health facility infrastructure, and the prevalence of detrimental cultural practices [13,17].

Our findings show that MMR is significantly influenced by key factors, such as maternal age at first birth, birth intervals, teenage motherhood, obesity, and HIV prevalence, all of which were identified as strong predictors of maternal mortality. Therefore, it strongly advocates for a comprehensive multidimensional approach to enhance maternal health care, surpassing the scope of purely medical interventions [7,18,19].

We also show that high fertility rates and low birth intervals have positive and negative Pearson's correlation with maternal mortality ratios, as they play a pivotal role in determining a woman's likelihood of conception and subsequent vulnerability to maternal mortality; thereby amplifying the need for improving interventions toward uptake of modern contraception. Notably, the inclusion of the uptake of modern contraception as a predictive variable was omitted from the model due to its strong collinearity with these aforementioned predictors.

The median age at first birth, along with the prevalence of thinness, significantly influences the probability of pregnancy and childbirth complications. These factors intricately impact a mother's capacity to sustain a healthy pregnancy to full term and facilitate a complication-free delivery. It is noteworthy that these predictors exhibit strong Pearson correlations with the prevalence of teenage motherhood, alongside other indicators pertaining to maternal nutritional well-being such as obesity and stunting (short stature). Moreover, the prevalence of HIV infection among women serves as a critical predictor reflecting infectious diseases that detrimentally affect maternal health [20]. Similarly, the occurrence of physical violence, particularly during pregnancy, signifies a cultural practice intertwined with women's empowerment, potentially elevating the risk of maternal complications [17]. These findings are in cohesion with previous research findings on indicators of maternal mortality [21,22].

Our model supports findings from a study with a similar design in Ethiopia [22]. However, our approach employed machine learning that extends usage to non-technical users due to its automation ability [9,12,14].

These predictors were effectively combined and harmonized to construct a robust model capable of precisely estimating MMR rates in both Kenya and its sub-populations. The model achieved a commendable accuracy level (R2) of 75.4%, which is a higher predictive accuracy as compared to previous statistical versions [22]. The examination of residuals, coupled with the juxtaposition of DHS estimates and model projections through a box and whisker plot, indicates that the model generated notably dependable estimates as compared to the national surveys. These estimates exhibit characteristics such as a mean closely aligned with the median and a narrower interquartile range with a diminished presence of outliers.

The application of the model to ascertain both national and subnational MMR values held significant importance due to the relatively dated nature of the most recent estimates, which were released nearly four years ago (2019 census). Furthermore, the absence of MMR estimates in the 2022 DHS publication created a substantial gap in vital information needed by stakeholders of maternal health in Kenya. This data plays a pivotal role in guiding targeted interventions and judicious resource allocation for the forthcoming five years when we anticipate having another DHS survey.

The 2014 DHS survey revealed a surge in maternal mortality within the marginalized counties of northern Kenya such as the Mandera, Marsabit, Turkana, and Isiolo Counties. This distressing situation prompted non-governmental organizations and the devolved county government at the time to address the crisis. For instance, Mandera County recorded an alarming estimated MMR of 3780 deaths per 100,000 live births. These interventions have made the previous dire situation significantly better, with the MMR dropping to an estimated 385 deaths per 100,000 live births based on the 2019 census estimates.

Moving forward, the model's MMR projections for 2022 indicate a national MMR of 367 (95% CI 154-579) deaths per 100,000 live births. Notably, this figure demonstrates minimal disparity from the 2014 DHS estimate of 362 deaths per 100,000 live births. Intriguingly while marginalized counties like Mandera exhibited improvement with a model-estimated MMR of 819 deaths per 100,000 live births, the lack of progress in the national average suggests the persistence of certain maternal health challenges. This circumstance could be influenced by a combination of ongoing maternal deaths compounded by the impact of the devastating COVID-19 pandemic that struck the country in 2020 [23,24].

The model's estimates reveal a concerning maternal mortality rate in marginalized counties, specifically Marsabit, Turkana, Tana-river, and Samburu, which exhibit elevated MMR values, ranging from 1000 to 1800 deaths per 100,000 live births. This situation is associated with inadequate health facility infrastructure, coupled with poverty and lack of education, in these counties [25]. Allocation of resources to improve access to health facility services, such as ambulances to increase mobility, and mobile clinics would significantly reduce MMR in these regions as well as the national MMR. Kisumu and Homa-Bay Counties follow with estimated MMRs of 859 and 642 deaths per 100,000 live births, respectively. This situation is closely related to an increase in the prevalence of HIV infections among women, particularly pronounced in counties proximal to Lake Victoria, with rates of 25% in Homa-Bay County and 21% in Kisumu County [20,26]. The model suggests allocation of resources on maternal HIV control and support of HIV-infected mothers in this region would significantly improve maternal health outcomes in the lake region.

Study limitations

Some limitations were noted for this study. First, the model depends on periodic DHS and census data, which may not reflect real-time maternal health changes and, therefore, limits the model's ability to respond to real-time maternal health crises. Second, the model does not account for unforeseen factors like pandemics or political instability that could impact maternal mortality trends. Future studies could enhance accuracy by integrating real-time data and incorporating dynamic variables such as healthcare funding or crisis response.

## Conclusions

Kenya's trajectory in reducing the national MMR remains suboptimal, marked by the significant variations observed across its counties. This disparity is further complicated by a lack of comprehensive data to guide effective intervention strategies. A potential solution lies in the advancement of artificial intelligence (AI) tools capable of predicting MMR and dynamically monitoring the outcomes of interventions. Such tools possess the capability to expedite the realization of sustainable development goal (SDG) 3.1, which targets maternal health improvement. The integration of predictive models that guide interventions and resource allocation will significantly contribute to improving maternal health care.

Our study not only predicts maternal mortality rates but also provides actionable insights for maternal health programs in Kenya. Identifying regions with the highest MMR can guide resource allocation and targeted interventions, for instance, counties with elevated MMR could benefit from increased healthcare funding and focused maternal health initiatives.
